# Factors associated with chronic pain in patients with bipolar depression: a cross-sectional study

**DOI:** 10.1186/1471-244X-13-112

**Published:** 2013-04-15

**Authors:** Inmaculada Failde, Maria Dueñas, Luis Agüera-Ortíz, Jorge A Cervilla, Ana Gonzalez-Pinto, Juan A Mico

**Affiliations:** 1 Department of Preventive Medicine and Public Health, University of Cádiz, Avda Ana de Viya 52, Cádiz 11009, Spain; 2 Psychiatry Department, University Hospital 12 de Octubre, Complutense University, Madrid, Spain; 3 Centro de Investigación Biomédica en Red de Salud Mental - CIBERSAM, University Hospital 12 de Octubre, Complutense University, Madrid, Spain; 4 Department of Psychiatry, University of Granada, Granada, Spain; 5 Centro de Investigación Biomédica en Red de Salud Mental - CIBERSAM, Hospital Universitario San Cecilio, Granada, Spain; 6 Centro de Investigación Biomédica en Red en Salud Mental - CIBERSAM, Hospital Santiago Apóstol, University of the Basque Country, Vitoria, Spain; 7 Department of Neuroscience, Pharmacology and Psychiatry, School of Medicine, University of Cádiz, Cadiz, Spain; 8 Centro de Investigación Biomédica en Red de Salud Mental - CIBERSAM, University of Cádiz, Cádiz, Spain

**Keywords:** Bipolar depression, Chronic pain

## Abstract

**Background:**

While pain is frequently associated with unipolar depression, few studies have investigated the link between pain and bipolar depression. In the present study we estimated the prevalence and characteristics of pain among patients with bipolar depression treated by psychiatrists in their regular clinical practice. The study was designed to identify factors associated with the manifestation of pain in these patients.

**Methods:**

Patients diagnosed with bipolar disorder (n=121) were selected to participate in a cross-sectional study in which DSM-IV-TR criteria were employed to identify depressive episodes. The patients were asked to describe any pain experienced during the study, and in the 6 weeks beforehand, by means of a Visual Analogical Scale (VAS).

**Results:**

Over half of the bipolar depressed patients (51.2%, 95% CI: 41.9%–60.6%), and 2/3 of the female experienced concomitant pain. The pain was of moderate to severe intensity and prolonged duration, and it occurred at multiple sites, significantly limiting the patient’s everyday activities. The most important factors associated with the presence of pain were older age, sleep disorders and delayed diagnosis of bipolar disorder.

**Conclusions:**

Chronic pain is common in bipolar depressed patients, and it is related to sleep disorders and delayed diagnosis of their disorder. More attention should be paid to study the presence of pain in bipolar depressed patients, in order to achieve more accurate diagnoses and to provide better treatment options.

## Background

The link between unipolar depressive disorder and pain has been investigated extensively in a number of epidemiological [1,2], clinical [3] and biological studies [4]. Accordingly, it has been demonstrated that a significant proportion of patients with unipolar depression experience pain, the intensity of which increases with the severity of the depressive symptoms [3]. A significant impact of pain on the course of depressive disorders has also been described, whereby patients that experience pain are more prone to chronic depression [5], and pain is a risk factor for a poorer therapeutic response [6].

Despite the association demonstrated between depression and pain, little is known about the relationship between pain and bipolar depression (BD), or its therapeutic consequences. A link between BD and pain has been proposed and indeed, BD is associated with an increased prevalence of migraine [7]. Indeed, migraine has been proposed as a trait of the bipolar spectrum in unipolar depressed patients [8]. Moreover, co-morbid migraine appears to be associated with poor outcomes in BD [9] and an increased prevalence of headaches during bipolar depression was recently related to increased rejection sensitivity during depression [10]. There is clinical evidence that a variety of pain conditions are common in BD [11], and that BD patients are significantly more likely than normal individuals to suffer from at least moderate pain [12]. However, there is still little data regarding the specific type of pain experienced by BD patients, including information regarding its intensity, localization and time course, and how this may modify the prognosis of this disorder and its treatment.

Better understanding the relationship between BD and pain is also vital to control the potentially risky pharmacological side-effects when treating pain in bipolar depressed patients. For example, chronic pain is frequently treated with antidepressants [13,14] which are known to predispose BD patients to manic switches and to increase the risk of suicide, particularly when administered in the absence of a mood stabilizer [15,16]. Anticonvulsants have analgesic properties [17] and they are commonly prescribed to patients with chronic pain (*i.e.,* neuropathic pain). However, while valproate, carbamazepine and lamotrigine in particular were thought to be useful to treat mania and bipolar depression, and in preventing relapses [18], these drugs have recently been associated with suicide attempts [19]. Likewise, there is conclusive evidence that due to a pharmacokinetic analgesic interaction, non-steroidal anti-inflammatory drugs can increase serum lithium levels, diminish renal lithium clearance and possibly induce lithium toxicity [20].

In relation to pain treatment, some analgesic opioids and other classical analgesics have been shown to have an important mood-altering effects on BD patients, increasing the risk of mania [21-23]. In particular, tramadol is an atypical opiate that inhibits serotonergic and noradrenergic reuptake and that is associated with intrinsic antidepressant-like properties [24]. However, this analgesic enhances the risk of inducing mania, probably due to its effects on the serotonergic system, although the potential role of noradrenaline cannot be ruled out (tramadol also inhibit the reuptake of this monoamine). Nevertheless the association of tramadol with Selective Serotonergic Reuptake Inhibitors (SSRIs) is clearly contraindicated, as is that of other antidepressants that block noradrenergic reuptake.

Chronic pain has recently been linked with an increased risk of suicide among individuals with mental disorders [25]. Given that BD is a major risk factor for suicide, the need for proper pain assessment in BD patients is more than justified [26]. Such procedures may be particularly useful for mental health practitioners for whom the treatment of pain is not part of their usual clinical practice, even though their patients may be prescribed antidepressants and/or anticonvulsants to manage chronic pain.

The aim of the present study was to evaluate the prevalence and characteristics of pain experienced by bipolar depressed patients treated by psychiatrists in their regular clinical practice, and to identify possible factors associated with the presence of pain in these patients.

## Methods

### Patient sample

Subjects were selected from among the participants in a multi-centre, cross-sectional study carried out on a representative sample of Mental Health Care Centres in Spain published elsewhere [27]. The sample included patients over 18 years of age who visited their psychiatrist for the first time and who were diagnosed with depression according to the *Diagnostic and Statistical Manual of Mental Disorders Fourth Edition Text Revision* (DS-IV-TR) criteria. In this study, 121 patients diagnosed with bipolar disorder currently suffering a depressive episode, and who were mentally and physically able to participate in the study, were analyzed. All the patients provided written informed consent before their inclusion in the study. Moreover, the study was carried out in accordance with the Helsinki Declaration, and the standard working procedures and protocols were approved by the Clinical Research Ethics Committee at the Clinic Hospital in Barcelona, ensuring adherence to the norms of good clinical practice.

### Instruments and variables

Interviews were performed at psychiatric out-patient centres and depression was confirmed on the basis of the diagnostic criteria of the DSM-IV-TR. The presence of anhedonia, loss of energy, sleep disorders, depressive mood, diminished concentration, change of body weight/appetite, feelings of guilt, psychomotor changes and suicidal ideation were assessed by the psychiatrist, and the intensity of depression was determined using the 17-item Hamilton Depression Scale validated in Spanish [28]. A Maier sub-scale score was extracted from the Hamilton Depression Scale (items 1, 2, 7, 8, 9 and 10) to measure the core symptoms of depression [29]. Information related to the duration of the current depressive episode and any prior history of depression was also collected.

The patients’ socio-demographic variables were recorded (age, sex, educational level and social status), and these patients were questioned about the presence of pain at the time of the study and during the preceding 6 weeks. In cases where pain was experienced, the intensity, duration and location of the pain were recorded. To ensure the clear-cut presence of painful symptoms, only patients with pain intensity over 40 on the Visual Analogue Scale (VAS) were considered (over a range of 0–100, where 0 represents no pain and 100 the worst pain possible [30]). Patients with pain were divided into two groups: subjects with pain of known aetiology, and those in whom the aetiology was unknown or only partially explained.

The interference of pain with the patients’ everyday activities in the week prior to the study was also assessed using a VAS, with a score of 0 representing no interference and 100 representing total incapacity. The data collection forms were further monitored centrally to check and correct any missing data or inconsistencies when possible.

### Statistical analysis

In the descriptive analysis, the absolute frequency, mean, median and the dispersion measurements were calculated for the qualitative and quantitative variables. The prevalence (± 95% CI) of pain was calculated, and the crude and adjusted odds ratios (ORs) were used to analyze the factors associated with pain. For this purpose, a logistic regression model was drawn up in which the outcome variable was the presence or absence of pain, including the following variables in the model: gender, age, marital status, Hamilton scale score, Maier subscale score, bipolar disorder with/without late bipolar diagnosis (previous diagnosis of other type of depression), and specific symptoms of depression (anhedonia, loss of energy, sleep disorders, depressive mood, diminished concentration, change of body weight/appetite, feelings of guilt, psychomotor change, suicidal ideation). A subset of the variables that best predicted the presence or absence of pain was selected using the Hosmer-Lemeshow test to identify the best model. Some interaction terms were also introduced into the model.

## Results

### Population characteristics

**Figure 1:**
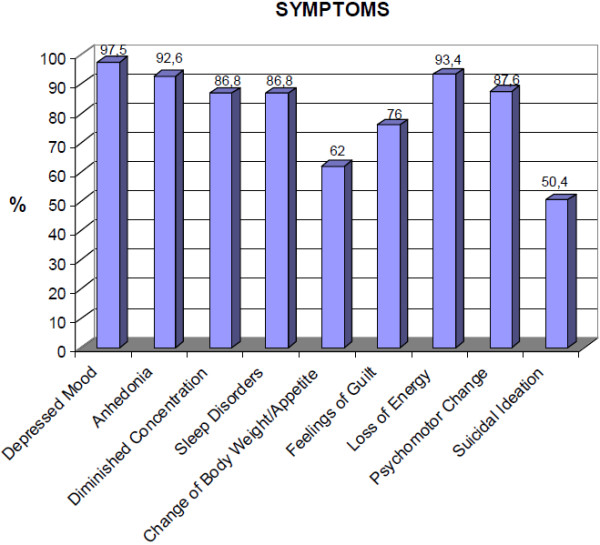
Prevalence of depressive symptoms in the studied patients.

The average age of the 121 participants was 50.7 years (SD 12.3), of whom 62.2% were female, 75.2% had completed primary or secondary education and 52.9% lived with their partner (Table 1). The mean number of previous depressive episodes experienced (including the current episode) was 4.28 (SD 3.08; median=3) and the median duration of the current depressive episode was 7 months (P25=5.5; P75=15). The mean score on the Hamilton 17 scale was 25.2 (SD 7.6) and the mean Maier score was 11.9 (SD 3.8: Table 1). The most prevalent depressive symptoms were depressed mood, loss of energy and anhedonia. Suicidal ideation was reported by 50.4% of patients (Figure 1).

**Table 1:** Characteristics of bipolar depressed patients

***Sociodemographic variables***		**N (%)**
*Sex* (N=119)	Male	45 (37.8)
	Female	74 (62.2)
*Age* (N=113)	Mean (SD)	50.69 (12.3)
*Age groups* (N=113)	<40	19 (16.8)
	40–49	34 (30.1)
	50–59	36 (31.9)
	60–69	15 (13.3)
	≥70	9 (8.0)
*Educational Level* (N=119)	Illiterate: No educational level completed	10 (8.4)
	Primary	59 (49.6)
	Secondary	31(25.6)
	University	19 (15.7)
*Marital status* (N=119)	Living with a partner	63 (52.9)
	Divorced/Separated	24 (20.2)
	Single	20 (16.5)
	Widow(er)	12 (9.9)
***Clinical variables***		
*Number of depressive episodes* (N=111)	Mean (SD)	4.28 (3.08)
	Median (P25;P75)	3.00 (2.0;6.0)
*Duration of the current depressive episode* (Months, N=29)	Mean (SD)	11.48 (9.68)
	Median (P25,P75)	7.00 (5.5,5.0)
*Hamilton Scale score* (N=121)	Mean (SD)	25.21 (7.6)
*Maier Scale score* (N=116)	Mean (SD)	11.98 (3.8)
*Diagnostic group* (N=121)	Bipolar disorder only	85 (70.2)
	Bipolar and other depressive disorders	36 (29.8)
*Pain* (N=121)	No	59 (48.8)
	Yes	62 (51.2)
*Duration of pain (Months)* (N=20)	Mean (SD)	62.5 (90.9)
	Median (P25,P75)	23 (6.0,90.6)
*Intensity of pain* (N=62)	Mean (SD); (Min,Max)	67.5 (14.9); (41,100)
	Median (P25,P75)	66.5 (56;78.5)
*Interference of pain in the activities of daily living* (N=62)	Mean(SD); (Min,Max)	67.7 (21.2); (0,100)
	Median (P25,P75)	67.9 (57.8,82.3)
*Pain location* (N=62)	Head	41 (66.1)
	Neck	41 (66.1)
	Back	46 (74.2)
	Limbs	42 (67.7)
	Joints	40 (64.5)
*Numbers of locations* (N=61)	Mean(SD)	3.44 (1.46)
	1	8 (13.1)
	2	10 (16.4)
	3	12 (19.7)
	4	9 (14.8)
	5	22 (36.1)
*Known Aetiology of pain* (N=57)	No	27 (47.4)
	Yes	30 (52.6)

*SD*, standard deviation; *P25*, 25th percentile; *P75*, 75th percentile.

The percentage of patients with BD as the sole diagnosis was 70.2%, while 29.8% were diagnosed with BD after having been diagnosed with other types of depression (30 major depression, 3 depression induced by physical disorders, 1 depression induced by illegal drugs and 2 dysthymia: Table 1).

### Prevalence and characteristics of pain

The prevalence of pain in the patients with BD was 51.2% (95% CI: 41.9%, 60.6%). There was a tendency for this prevalence to be higher in the female population (66.1% vs 33.9%, *χ*^2^=0.856, p=0.355), and in patients between 40 and 59 years of age (62% vs. 38.1%, *χ*^2^=5.267, p=0.261), although these differences were not significant. The characteristics of the pain experienced are shown in Table 1. The average duration of pain was 68.9 months (median, 23 months; P25=6.0; P75=90.6), the mean intensity on the VAS was 67.5 (SD 19.4) and the most common location of pain was in the back (74.2%), while on average pain was located at 3.44 sites (SD 1.5). The aetiology of the pain was known and specified in 30 patients (Table 1), the most common cause being a musculoskeletal pathology (75%). The average score in the scale measuring inability to perform everyday activities due to pain was 67.7 (SD 21.2).

### Factors associated with pain

Certain factors were associated with the pain experienced by patients suffering from BD (Table 2). The adjusted model identified divorce or separation and diagnosis with another depressive disorder prior to the diagnosis of BD (delayed diagnosis of bipolar disorder) as risk factors independently associated with the presence of co-morbid pain in these patients (Table 3). Likewise, the existence of sleep disorders produced an Odds Ratio of 3.41, although there was no significant association with the existence of pain. The probability of experiencing pain rose by 2% each year that the patient’s age increased, although this effect did not reach statistical significance in this population (Table 3).

**Table 2:** Factors associated with pain in bipolar depressed patients

**Variable**	**Label**	**OR**	**CI 95%**	**p-value**
Sex (N=119)	Male *			0.355
	Female	1.42	(0.68;2.99)	
Educational Level (N=119)	Illiterate: No educational level completed*			0.523
	Primary	1.27	(0.33;4.86)	
	Secondary	1.21	(0.29;5.06)	
	University	0.58	(0.12;2.75)	
Marital status (N=119)	Living with a partner *			0.098
	Divorced/Separated	0.69	(0.27;1.79)	
	Single	0.97	(0.35;2.65)	
	Widow(er)	4.84	(0.98;23.91)	
Age (N=113)		1.03	(1.00;1.07)	0.034
Diagnostic group (N=121)	Bipolar disorder only *			0.069
	Bipolar and other depressive disorders	2.09	(0.94;4.66)	
Depressed Mood (N=121)	Yes	2.14	(0.19;24.25)	0.527
Anhedonia (N=121)	Yes	0.83	(0.21;3.25)	0.787
Diminished Concentration (N=121)	Yes	1.41	(0.49;4.08)	0.520
Sleep Disorders (N=121)	Yes	2.61	(0.85;8.05)	0.083
Change of body weight/appetite (N=121)	Yes	1.25	(0.59;2.60)	0.556
Feelings of worthlessness or guilt (N=121)	Yes	1.69	(0.72;3.93)	0.222
Loss of Energy (N=121)	Yes	3.39	(0.68;17.55)	0.117
Psychomotor Activity changes (N=121)	Yes	1.23	(0.42;3.64)	0.705
Suicidal Ideation (N=121)	Yes	0.74	(0.36;1.52)	0.412
Number of depressive episodes (N=111)		0.96	(0.85;1.08)	0.492
Duration of the current depressive episode (Months, N=29)		1.05	(0.95;1.16)	0.300
Hamilton Scale score (N=121)		1.04	(0.99;1.09)	0.118
Maier Subscale score (N=116)		1.05	(0.96;1.16)	0.301

* Reference Category.

**Table 3:** Logistic regression model of the variables associated with pain in bipolar depressed patients

**Variable**	**Label**	**B**	**Adjusted OR**	**CI 95%**	**p-value**
**Marital status**	Living with a partner *				
	Divorced/separated	−1.25	0.29	(0.09; 0.93)	0.038
	Single	0.24	1.28	(0.39; 4.08)	0.681
	Widow(er)	2.15	8.58	(0.86; 85.34)	0.067
**Diagnostic group**	Bipolar disorder only *				
	Bipolar and other depressive disorders	1.26	3.51	(1.22; 10.15)	0.020
**Sleep Disorders**	No*				
	Yes	1.23	3.41	(0.89; 13.09)	0.074
**Age (years)**		0.020	1.02	(0.98; 1.06)	0.341

Hosmer-Lemeshow=6.695; p-value=0.570.

* Reference Category.

## Discussion

The results obtained in this study reveal a high prevalence of pain in patients diagnosed with BD and in particular, approximately 2/3 of the women with BD experienced concomitant pain. The intensity of pain was moderate or severe, of prolonged duration and it arose at multiple sites, resulting in a significant limitation on everyday activities. The most important factors associated with the presence of pain in our study were older age, being separated or divorced, having a prior diagnosis of other types of depression, and the existence of sleep disorders.

Bipolar disorders are associated with increases in functional decline, mortality and healthcare costs. Moreover, despite the efficacy of some treatments, the outcomes for these patients currently remain suboptimal [31,32]. Physical co-morbidity is frequent in BD patients [33], and pain is one of the most frequent and important causes of comorbid physical symptoms in a variety of mental illnesses. The present findings reveal a high prevalence of pain in BD patients, which is important given the paucity of data of this co-morbidity in this patient group and the potential adverse side-effects associated with pain management in BD patients [16,19]. Indeed, there is an increased risk of attempted suicide in patients with depression and chronic pain [34], highlighting the need for routine evaluation and monitoring of suicidal behaviour in these patients [35]. The prevalence of chronic pain in the BD patients studied may be associated with a higher risk of committing suicide. While no such direct correlation was found in our study, it should be born in mind that the sum of the two risk factors could enhance this possibility. However, to our knowledge no large clinical studies have been performed to explore this possibility.

In recent years, several studies have investigated the association between depression and chronic pain [1], demonstrating that depression and pain can trigger and perpetuate one another, due to overlapping neural and emotional alterations [36]. The interference produced by pain in BD patients has been explored recently [12], and although the specific factors associated with the presence of pain were not investigated, age, co-morbid anxiety disorders and co-morbid medical conditions appeared to be independently associated with pain interference.

We found that almost 30% of BD patients have suffered other forms of depression previously. In relation with this finding it is interesting that a prior diagnosis of another type of depression (particularly major depression) was related to a delay in the accurate diagnosis of bipolar disorder [37]. Interestingly, the prior diagnosis of another type of depression was a factor associated to suffering pain in our study and thus, we assume that pain may well be a relational factor that influences the delayed diagnosis of bipolar disorder. However, the transverse nature of our study does not enable us to probe this hypothesis.

It was recently suggested that inter-episode REM sleep, slow-wave sleep and stage 2 sleep are correlated with a predisposition to manic and depressive symptoms [38], and certain sleep parameters have been linked to the mechanisms maintaining illness in BD [38]. Likewise, chronic pain has been associated with sleep difficulties, which is probably a reciprocal relationship [39]. Because chronic pain is frequently co-morbid with psychiatric disorders, it is unclear to what extent chronic pain itself is associated with these conditions. A large community-based epidemiological survey [40] recently reported significant associations between chronic pain and sleep problems. As expected, these associations were stronger for chronic pain that was co-morbid with psychiatric disorders. Here, the presence of sleep disorders in bipolar patients was a risk factor for pain, and while our findings are consistent with these earlier results [40], they should be interpreted with caution due to the small sample size, and they must be validated by more extensive studies in the future. However, our results should be taken into consideration when planning treatment for BD patients with co-morbid pain. Interestingly, targeted therapy for insomnia was previously shown to improve pain symptoms and may also lead to improvements in associated mental disorders [41].

One limitation of the present study was the small size of the sample, and the fact that some factors associated to pain (OR > 1) were of borderline statistical significance. Likewise, the cross-sectional nature of the survey does not allow us to obtain information regarding the direction of the causality between pain and BD. Therefore, we can only speculate as to how the proposed association between pain and BD is established. It has been suggested that certain pain conditions, like migraine [42], share a common predisposition with some psychiatric disorders [43]. However, more follow-up studies will be necessary to further our understanding of these relationships. Moreover, our study did not assess the presence of anxiety or non-psychiatric co-morbidity frequently observed in bipolar patients [12]. These limitations were offset by several key strengths, including our use of a dataset collected from a wide range of psychiatric centres and the objective approach to pain assessment.

## Conclusions

In summary, we found a high prevalence of moderate to severe intensity pain in bipolar patients, which is persistent and significantly affects the patients’ ability to carry out everyday activities. We propose that greater attention should be paid to the presence of pain in bipolar patients, as well as to other factors that could influence their clinical development, such as sleep disorders and the presence of other forms of depression.

## Abbreviations

VAS: Visual analogical scale; BD: Bipolar depression; SSRIs: Selective serotonergic reuptake inhibitors; DS-IV-TR: Diagnostic and statistical manual of mental disorders fourth edition text revision; ORs: Odds ratios.

## Competing interests

The authors declare that they have no competing interests.

## Authors’ contributions

FI, GPA and MJA performed the literature searches and wrote the first draft. DM undertook the statistical analysis and wrote the first draft. FI, AOL, CJA and MJA designed, planned and coordinated the study. All the authors contributed to and have approved the final version of the manuscript.

## Pre-publication history

The pre-publication history for this paper can be accessed here:

http://www.biomedcentral.com/1471-244X/13/112/prepub
